# Association between gastroesophageal reflux disease and depression disorder: A protocol for systematic review and meta-analysis

**DOI:** 10.1097/MD.0000000000022696

**Published:** 2020-10-23

**Authors:** Yu Liu, Panpan Zhou, Shixiong Zhang, Huiqing Wu, Zeqi Yang, Miaochan Xu, Shaowei Liu, Yangang Wang

**Affiliations:** a School of Pharmacy, Hebei University of Chinese Medicine; b Department of Gastroenterology, The First Affiliated Hospital of Hebei University of Chinese Medicine, Shijiazhuang, Hebei, P.R. China.

**Keywords:** association, depression disorder, gastroesophageal reflux disease, meta-analysis

## Abstract

**Background:**

This study will systematically synthesize the evidence on the potential association between gastroesophageal reflux disease (GERD) and depression disorder (DD).

**Methods:**

We will search the following electronic bibliographic databases: PubMed/MEDLINE, Embase, Cochrane Library, Web of Science, the Chinese Bio Medical Literature Database, China National Knowledge Infrastructure (CNKI), the China Science and Technology Journal database (VIP) and Wanfang Data. In addition, ongoing trials will be retrieved from the WHO ICTRP Search Portal, the Chinese Clinical Trial Register and The Clinical Trials Register. Articles related to gastroesophageal reflux disease and depression will be searched. And language and time will be unlimited.

**Results:**

The study will afford additional insight into the investigation the association between GERD and DD.

**Conclusions:**

The results of this study will provide helpful evidence to explore the association between GERD and DD.

**Registration number:**

INPLASY202090026.

## 1 Introduction

According to the Montreal definition, gastroesophageal reflux disease (GERD) is defined as a disease which is associated with troublesome symptoms and/or complications on account of reflux of stomach contents into the esophagus. Most patients with GERD presents with heartburn and effortless regurgitation.^[1]^ GERD is one of the most prevalent gastrointestinal disorders globally, showing an increasing prevalence in several developing countries, but there is considerable geographic variation.^[2]^ The result of a direct pairwise comparison and NMA shows that esophageal reflux monitoring or endoscopy is superior to other diagnostic tests for GERD, which is in accordance with the recommendation in current guidelines.^[3,4]^ It has been reported that the pooled prevalence of at least weekly GERD symptoms reported from population-based studies worldwide is approximately 13%, but there is considerable geographic variation.^[5]^ The disease has a chronic course that leads to a significant decline in the quality of life of patients and is associated with a high economic burden worldwide.^[6]^

Major depressive disorder, also known as depression, is a common neuropsychiatric disorder that affects more than 300 million people of all ages and is one of the leading causes of global disease burden.^[7,8]^ It is a very common medical condition that is associated with a wide range of emotional, cognitive, and physical symptoms. Depressive disorders involve all major bodily functions, mood, and thoughts, affecting the ways in which a depressed individual, eats and feel about themselves, and thinks. Without treatment, depressive symptoms can last for weeks, months, or a life-time. Measured by years that people spend disabled with depression, it is the biggest blight on human society—bar none.^[9–11]^

Many studies have investigated the relationship between functional gastrointestinal disorder and psychological factors.^[12–14]^ A close relationship has been established between the brain and the gastrointestinal tract. For example, stress and emotions can affect gastrointestinal function, as well as the occurrence of gastrointestinal symptoms and disease.^[15]^ Likewise, the state of the gastrointestinal organs may affect a persons emotional status. Psychological factors may influence the severity of a functional gastrointestinal disorder by affecting the perception of pain through an action on the gut-brain axis—a concept that is also applicable to patients with GERD.^[16]^To date, a few studies describing GERD have shown that psychological factors, particularly anxiety and depression, play an important role in patients with GERD.^[17–19]^ However, a comprehensive explication of this association had not yet been given. Thus, this study aims to systematically investigate the association between GERD and DD.

## 2 Methods

### 2.1 Objective

This study will aim to explore the association between GERD and DD systematically and comprehensively.

### 2.2 Study registration

We have registered this study on INPLASY202090026. It has been reported according to the guideline of preferred reporting items for systematic reviews and meta-analysis protocol statement. And we followed the PRISMA-P guidelines.^[20,21]^

### 2.3 Inclusion criteria for study selection

#### 2.3.1 Type of studies

All potential case-controlled studies will be included, which identified the association between GERD and DD, regardless language and publication status limitations.

#### 2.3.2 Type of participants

The patients with clinically diagnosed GERD, regardless of race, gender, and age.

#### 2.3.3 Type of exposures

Exposure reviewed is the diagnosis of DD which is defined by each individual study. And the comparator is no diagnosis of GERD.

#### 2.3.4 Outcomes

Relative risk of DD in people with GERD compared to people without GERD.

### 2.4 Search methods

We will search the following electronic bibliographic databases: PubMed/MEDLINE, Embase, Cochrane Library, Web of Science, the Chinese Bio Medical Literature Database, China National Knowledge Infrastructure (CNKI), the China Science and Technology Journal database (VIP) and Wanfang Data. In addition, ongoing trials will be retrieved from the WHO ICTRP Search Portal, the Chinese Clinical Trial Register and The Clinical Trials Register. Articles related to gastroesophageal reflux disease and depression will be searched. And language and time will be unlimited.

### 2.5 Search strategy

Two authors will screen the titles and abstracts of the all records retrieved in above electronic databases independently to find potentially eligible reviews. According to the inclusion and exclusion criteria outlined above, the full texts of them will be retrieved for further identification. Any disagreement will be resolved by discussion or by consultation with a third author. The search strategy for PubMed is presented in Table 1 and the strategy will be modified upon the requirement of other databases.

**Table 1:**
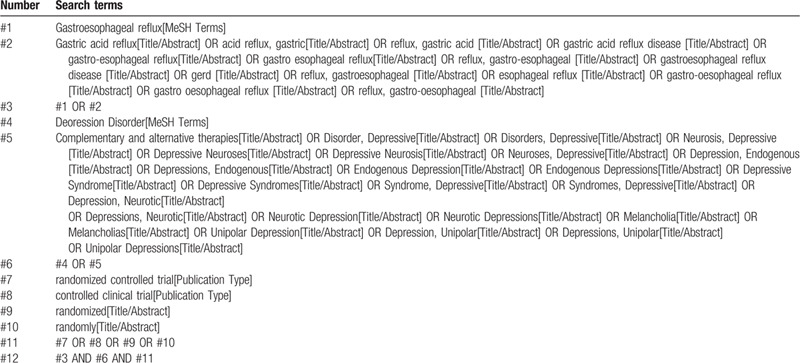
Search strategy used in PubMed database.

### 2.6 Data collection and analysis

#### 2.6.1 Study selection

Two reviewers will perform literature screening, study selection, and data extraction independently. The literature obtained will be imported into EndnoteX9 to screen the title and abstract, the duplications and studies failing to meet the pre-specified inclusion criteria will be excluded. After reading the full text of the remained literature and discussing within the group, the final included studies will be determined. The corresponding author of original RCT will be contacted when the full text is unavailable. Disagreements will be solved by consulting a third-party arbitrator or discussing within a group.

#### 2.6.2 Data extraction and management

Two authors will screen the titles and abstracts of the all records retrieved in above electronic databases independently to find potentially eligible reviews. According to the inclusion and exclusion criteria outlined above, the full texts of them will be retrieved for further identification. Any disagreement will be resolved by discussion or by consultation with a third author.

Data will be extracted by 2 reviewers independently using a pre-designed data extraction form. A third reviewer will validate data. The following data will be extracted: General information, Trial characteristics, Intervention(s) and control(s), Participants, Study methodology, Outcomes, Results, etc.

#### 2.6.3 Risk of bias in included studies

The methodological quality of eligible studies will be assessed by 2 review authors independently according to the the Cochrane Handbook for Systematic Reviews of Interventions. The following characteristics will be assessed: random sequence generation (selection bias), allocation concealment (selection bias), blinding of participants and personnel (performance bias), blinding of outcome assessment (detection bias), incomplete outcome data (attrition bias), selective reporting (reporting bias), other bias. Based on the assessments of the studies against these 7 domains, they will be classified as being of “low risk”, “high risk” or “unclear risk” of bias. Any disagreements will be resolved by discussion or discussed with another reviewer if necessary.

#### 2.6.4 Data analysis

Meta-analysis was conducted using Review Manager software (version 5.3). Odds ratio (OR) with 95% confidence intervals (CI) was reported for the dichotomous data, and mean differences (MD) with 95% CI for the continuous data. Statistical heterogeneity between studies was tested by calculating Higgins *I*^2^ values or using the χ^2^ test. *I*^2^ > 25%, *I*^2^ > 50%, and *I*^2^ > 75% were respectively defined to indicate moderate, substantial, and considerable heterogeneity. When the *P* value of χ^2^ test was <.1, an *I*^2^ test was carried out. If the *I*^2^ test showed a value >50%, a random effects model was carried out. Otherwise, a fixed effects model was carried out. A *P* value lower than.05 was considered to be statistically significant.

#### 2.6.5 Patient and public involvement

This is a meta-analysis study based on previously published data, so patient and public involvement will not be included in this study.

#### 2.6.6 Grading the quality of evidence

The Grading of Recommendations Assessment, Development and Evaluation (GRADE) guidelines will be utilized to grade the quality of evidence as very low, moderate, or high.

## 3 Discussion

This will be the first study to investigate the association between GERD and DD. It will systematically and comprehensively search electronic databases, and other literature sources to avoid missing potential studies. This study will summarize the most recent eligible studies of the association between GERD and DD. The findings of this study will provide evidence to judge the association between GERD and DD, which may benefit for the clinical practice and future studies.

## Author contributions

**Conceptualization:** Yu Liu, Yangang Wang.

**Data curation:** Yu Liu, Panpan Zhou.

**Formal analysis**: Shixiong Zhang.

**Funding acquisition**: Yangang Wang.

**Methodology:** Shaowei Liu, Zeqi Yang, Miaochan Xu.

**Resources:** Yangang Wang.

**Software:** Shaowei Liu, Zeqi Yang.

**Supervision**: Huiqing Wu.

**Writing – original draft:** Yu Liu.

**Writing – review & editing**: Yu Liu, Miaochan Xu.
